# Projecting health labor market dynamics for a health system in transition: planning for a resilient health workforce in Saudi Arabia

**DOI:** 10.1186/s12992-021-00747-8

**Published:** 2021-09-14

**Authors:** Tracy Kuo Lin, Tim A. Bruckner, Taghred Alghaith, Mariam M. Hamza, Mohammed Alluhidan, Christopher H. Herbst, Hussah Alghodaier, Adwa Alamri, Rana Saber, Nahar Alazemi, Jenny X. Liu

**Affiliations:** 1 Institute for Health & Aging, Department of Social and Behavioral Sciences, University of California, 490 Illinois St, CA 94158 San Francisco, USA; 2 Center for Population, Inequality, and Policy, University of California, Irvine, USA; 3 Saudi Health Council, Riyadh, Saudi Arabia; 4 World Bank, Washington, DC USA; 5 Division of Health Research, Lancaster University, Lancaster, UK

**Keywords:** Human resources for health, Health workforce planning, Labor market projections, Health system in transition, Saudi Arabia, Supply, Demand, Physicians, Nurses, Vision 2030

## Abstract

**Background:**

Health workforce planning is critical for health systems to safeguard the ability to afford, train, recruit, and retain the appropriate number and mix of health workers. This balance is especially important when macroeconomic structures are also reforming. The Kingdom of Saudi Arabia is moving toward greater diversification, privatization, and resiliency; health sectorreform is a key pillar of this transition.

**Methods:**

We used the Ministry of Health Yearbook data on the number of workers and health expenditures from 2007 to 2018 and projected health labor market supply and demand of workers through 2030, evaluated the potential shortages and surpluses, and simulated different policy scenarios to identify relevant interventions. We further focused on projections for health workers who are Saudi nationals and health worker demand within the public sector (versus the private sector) to inform national objectives of reducing dependency on foreign workers and better deploying public sector resources.

**Results:**

We projected the overall health labor market to demand 9.07 physicians and nurses per 1,000 population (356,514) in 2030; the public sector will account for approximately 67% of this overall demand. Compared to a projected supply of 10.16 physicians and nurses per 1,000 population (399,354), we estimated an overall modest surplus of about 42,840 physicians and nurses in 2030. However, only about 17% of these workers are estimated to be Saudi nationals, for whom there will be a demand shortage of 287,895 workers. Among policy scenarios considered, increasing work hours had the largest effect on reducing shortages of Saudi workers, followed by bridge programs for training more nurses. Government resources can also be redirected to supporting more Saudi nurses while still ensuring adequate numbers of physicians to meet service delivery goals in 2030.

**Conclusion:**

Despite projected overall balance in the labor market for health workers in 2030, without policy interventions, severe gaps in the Saudi workforce will persist and limit progress toward health system resiliency in Saudi Arabia. Both supply- and demand-side policy interventions should be considered, prioritizing those that increase productivity among Saudi health workers, enhance training for nurses, and strategically redeploy financial resources toward employing these workers.

**Supplementary Information:**

The online version contains supplementary material available at 10.1186/s12992-021-00747-8.

## Background

Building an effective and efficient health system requires proactive planning for the health workers who are essential to its functioning. Decision-makers should dynamically engage in health workforce planning and should budget for the resources needed to safeguard the ability to afford sufficient health workers and to train, recruit, and retain the right number and appropriate mix of health workers. Generating evidence on the dynamics of the health labor market, including concepts of *labor market supply* (the number of workers produced and willing to work in the health labor market) and *labor market demand* (the financing and absorption capacity at the institutional or national level to recruit health workers) is critical for this planning [1].

Global health labor market supply and demand predictions suggest that many countries will likely experience shortages in the future (i.e., there will be more financing to recruit health workers than individuals willing to work as health workers), which in middle- and high-income countries will be driven by growth in the need-based demand for health workers [2]. However, these analyses make general assumptions and yield only aggregate trends across countries; they are insufficient for informing country-level workforce strategies.

Health workforce planning is especially critical and opportune when the larger political economy is transitioning from one that is dominated by specific industries or market sectors to a more diverse economy amid increasing globalization. The Kingdom of Saudi Arabia (KSA) has embarked on a National Transformation Program [3], which outlines 34 objectives across different government sectors in an effort to diversify its economy and raise the standard of living of its people. Transforming the health system forms one key pillar of this wide-ranging strategy and involves reforms to promote public health via a number of objectives: emphasizing prevention and primary care, improving equitable access to health services, reducing reliance on foreign health workers, and optimizing public sector resources while mobilizing those from the private sector [4].

To prepare the KSA health workforce for changing health system priorities, an assessment of the health workforce dynamics and composition is crucial. In 2019, there were approximately 300,000 physicians and nurses in the KSA, or about 7 to 9 physicians and nurses per 1,000 population [5]. However, Saudi nationals comprise only about one-third of this workforce; two-thirds are non-Saudi migrant health workers [6]. Epidemiological need-based modeling of health workers has estimated that the KSA will require between 1.64 and 3.05 physicians and nurses per 1,000 population to provide health services in 2030 [7].

However, it is unclear how these need-based estimates comport with the future availability (i.e., supply) and affordability (vis-à-vis demand) of health workers in 2030 given the labor market dynamics for health workers. In other words, how does the number of qualified workers available to work in the sector compare with the willingness of employers to hire these workers given their available resources [2]? More importantly for strategic planning purposes, how many of those available may be Saudi national workers in line with national workforce resiliency goals? It is equally essential to also consider how different policies may mitigate or exacerbate the resulting shortages (or surpluses) of health workers. A situation in which a labor shortage exists could call for policy solutions to increase the supply of health workers (production output). Examples of these policy solutions include improving retention through providing early career preparation and workplace support [8], delaying the workforce retirement age [9], and expanding medical [10] and nursing training programs [9].

To inform human resources for health planning in the KSA, we assess the current and projected (to 2030) health labor market supply and demand of health workers to determine any health labor market surpluses or shortages. We focus on physicians and nurses because of their central role in providing care for disease conditions prioritized in the KSA [7]. We also pay special attention to the supply of Saudi health workers and the demand for health workers in the public sector given the specific strategic direction of health reforms. Lastly, we simulate some different policy scenarios—increasing work hours, improving retention, providing continuing education for nurses, and reallocating health worker budget resources—to assess the relative impact of different policy options to inform priority setting. The findings provide insight on the status quo health labor market supply and demand, assuming no policy intervention, and the potential impact of different policies on supply and demand.

## Methods

We adopted an econometric modeling approach to project health labor market supply and demand for health workers, focusing on physicians and nurses, following previous methods used in the literature [2]. *Health labor market* supply reflects the number of health professionals with the appropriate skills and qualifications who are willing to take available jobs in the health sector [11]. *Health labor market* demand is the willingness of providers, such as the government or private employers, to recruit and absorb health workers into the health system. Both supply and demand models used historical data on the number of physicians and nurses and population size from 2007 to 2018 [12]. Demand modeling additionally used data on total Ministry of Health (MOH) expenditures and total governmental expenditures. Table A1 in Appendix A summarizes the details on data sources.

### Projecting Supply

We projected the supply of physicians and nurses for (1) the total health labor market and (2) by nationality (Saudi health workers versus foreign, migrant workers). Regional data on physicians and nurses (stratified by Saudi vs. foreign) employed by (1) the MOH in the public sector, and (2) by all employers in the private sector were available; regional data for physicians and nurses employed by non-MOH public agencies^2^ were not available. The MOH employs approximately 75 % of physicians and nurses working in the public sector, and accounts for the largest share (65 %) of governmental health expenditures in the KSA [13]. Therefore, despite limitations, these data captured the majority of public sector workers, and the resulting analyses provide insightful stratification across policy-relevant workforce characteristics. It should also be noted that data on physicians included a small portion (10 %) of dentists who could not be disaggregated. Data on nurses are discussed in more detail below.

Various econometric approaches can be used to project the supply of health workers into the future, each with advantages and disadvantages. A growth rate approach required the least amount of data but relied heavily on the functional form assumption. A moving average, or distributed lag model, gave more weight to more recent data but required that data be available for a continuous number of years. An autoregressive integrated moving average (ARIMA) model required that a long time series be available — ideally back to 1980 — with very few missing data points.

Given the available data, we projected the supply of workers based on the historical growth rate—a method used for this purpose in previous studies [2, 14]. We adopted a linear functional form and assumed that growth in health worker supply is exogenous and trends with time. Labor market inflows (e.g., from education sector outputs) and outflows (e.g., attrition, death) are thus implicitly captured by the model as trends in these factors are similarly assumed to remain constant. This model implies a relatively rigid labor market, which may be plausible for a health labor market characterized by limited competition in the short run. To project the supply of Saudi and foreign health workers separately, we similarly applied the growth rate method for each series. We adjusted supply projections upwards to account for non-MOH public sector workers by increasing the total by approximately 25 %—the share of the non-MOH workforce (as measured in 2018)—for all future years to obtain the total national supply of health workers and the subset who are Saudi nationals.

For our Saudi supply projections, we incorporated two additional adjustments to better reflect operational characteristics of the Saudi health workforce. First, we converted projected supply of workers to their full-time equivalent (FTE). Public sector health workers typically work five hours per day as compared to a full eight-hour workday. Therefore, we multiplied projected supply estimates for public sector workers (MOH and non-MOH) by 63 % (downward adjusted) to generate their FTE equivalency; we evaluated separately the impact of varying working hours on the supply of health workers using comparative statistics in analyses below.

Second, we adjusted the number of nurses downward to account for variation in scopes of work for different nursing sub-cadres. In the KSA, nurses with a four-year bachelor’s or more advanced degrees typically take a more difficult exam that qualifies them as nurse specialists, more involved in-patient care. This scope of practice is better aligned with international classifications for the nursing profession more generally. Data on nurses from the MOH aggregate all three types of nurses, and resulting projections thus overestimate the true supply of nurses who perform patient care duties. Therefore, we adjusted projected yearly number of all nurses downward by 67 % (the percentage of nurses in the KSA who have diploma degrees based on 2018 data) to capture only nurses with bachelor’s and advanced degrees.

The projections – estimated using the above methodology – provided a baseline, status quo labor market supply that assumed no policy change; the inflow and outflow factors remained constant over time. To consider potential impact of policy interventions, such as introducing training programs, we relaxed some of our model assumptions and modeled the effect of some alternative scenarios in the simulated policy scenario section.

### Projecting Demand

We projected the demand for physicians and nurses for (1) the overall health labor market and (2) also for just the public sector to estimate the number of workers that the government can afford. One can define *labor market demand* as the amount of financing available to absorb health workers into the health labor market. In the public sector, financial resources for health connect to the budget allocated to the wage bill, which in turn links to resources allocated to health overall. In the private sector, financial resources to recruit health workers stem from population demand for private health care and the willingness of consumers to pay for those services, the delivery of which requires health workers. The main notion of labor market demand is that health workforce purchasers, whether public or private, can recruit only as many health workers as they can afford (i.e., those for whom they have the budget) [11]. For a health system in transition, such as the one in the KSA, where both public and private sector employers are undergoing transformations, the variable demand in each sector may require distinct policy interventions.

We developed an economic model to predict future densities of physicians and nurses through 2030, drawing on past trends and correlations between economic drivers and worker densities [2, 14]. Our demand model assumes that there will be no changes in wages, skills mix, tasks, and productivity for health workers, health systems, or technology in the short run. Although wages are a key determinant of the quantity of labor employed, the model holds wage growth constant in order to assess the independent effects of aggregate spending patterns. In reality, differentials in wages and the producitivity of different health workers will affect the numbers of workers demanded—fewer workers may be needed at higher levels of productivity to deliver the same amount of services (all else being equal) and may thus command a higher salary. However, these factors are often static in the short term (e.g., with civil service compensation schedules) and may require large-scale implementation (e.g., of technologies that enhance productivity) to effect macroeconomic change.

We used available subnational economic data on health expenditures in the KSA as our main predictor variables. This approach contrasts with past studies, which relied on proxy indicators such as gross domestic product or national income [15–18] because of the lack of available data on health expenditures. However, such macroeconomic indicators and measures for the private sector (e.g., non-government expenditures on health, private out-of-pocket spending for health) are not available at the subnational level for the KSA. While this is a notable data limitation, labor demand mainly driven by public sector spending may appropriately portray the status quo situation in the KSA. As macroeconomic transition has only recently begun, government spending continues to dominate the market and private health expenditures have been relatively stable over the past 16 years [12].

We used per capita total government expenditures and per capita MOH expenditures on health to predictor health worker densities using the following model:

1 $$ \ln \left( health\ workers\  per\  1, 000\ {population}_{\mathrm{pt}}\right)={\displaystyle \begin{array}{c}{\upbeta}_{\mathrm{o}}{\upbeta}_1\ast \ln \left(\mathrm{MOH}\ \mathrm{health}\ \mathrm{expenditure}\ \mathrm{per}\ {\mathrm{capita}}_{\mathrm{pt}\hbox{-} 1}\right)\\ {}{\upbeta}_2\ast \ln \left(\mathrm{total}\ \mathrm{government}\ \mathrm{expenditure}\ \mathrm{per}\ {\mathrm{capita}}_{\mathrm{pt}\hbox{-} 1}\right)\\ {}+{\mu}_p+{\xi}_{pt}\end{array}} $$

where *µ*_*p*_ represents a vector of provincial fixed effects to account for time-invariant unobservable heterogeneity (that is, not captured by available data) across provinces (e.g., average distance to closest hospitals), ξ_pt_ is the disturbance terms, and β coefficients are unknown parameters to be estimated from the model. ξ_ct_ predictor variables were lagged to ensure the direction of causality and allow time for these factors to work through the economy to affect the labor market [2, 14, 17]. To identify the optimal correlation of lagged government expenditures (in total and for health) from 2009 to 2018 that most strongly relates to the historical density of physicians and nurses per 1,000 population, we used a stepwise approach (see Appendix B for detailed analytical steps and model fit). Then, based on the estimated coefficients of economic predictor variables and their forecasted values in future years, the model predicts the future density of physicians and nurses in each province. Note that we projected estimated future values of both public spending drivers in a separate exercise (see Appendix B). We then multiplied the predicted density for each cadre in each province-year by the future population estimates to calculate the number of health workers demanded per province.

Because historical public sector health worker data were available only for workers employed by the MOH, total estimated worker numbers were adjusted upwards to account for additional workers employed by non-MOH employers using the same procedure applied to aggregate supply estimates. This approach implicitely assumes that trends in private sector spending will continue despite not explicitly being specified in the model.

Following the above methodology, the labor market demand projections resulted in baseline, status quo estimates which assumed no policy intervention. To consider potential policy impact on labor market demand, we relaxed some of the assumptions and evalauted the influence of resource allocation in a simulated scenario below.

### Simulated Policy Scenarios

To simulate different policy options, we relaxed some of our model assumptions and modeled the effect of some alternative scenarios on estimated health workforce gaps. We used comparative statistics to quantify the independent effect of each hypothetical policy intervention in reducing shortages.

We examined three supply-side policies targeted toward workers who are Saudi nationals—increasing work hours, improving retention, and providing continuing education for nurses—and one demand-side option for reallocating health worker budgets; these policy interventions have been identified as potential policies by local government experts from the Saudi Health Council, the Saudi Commission for Health Specialties, and the MOH. Each policy may contribute to strategic plans for increasing the capacity of domestic workers, including illustrating how redirecting public sector demand resources may help to achieve this aim.

1. *Increasing working hours.* Higher productivity may translate to the ability to serve more patients or attend to more visits with the same number of health workers. We compared the baseline scenario that reflects the current five-hour workday common among Saudi workers (that is, 63 % productivity) with an eight-hour workday (100 % productivity).^3^
2. *Improving retention.* Delaying retirement age can increase the number of workers engaged in patient care by reducing the number of workers who would otherwise retire. The KSA population is aging—the population over age 60 is projected to increase from 3 % in 2010 to 10 % in 2035 [18]. We examined the impact of increasing retirement by 10 years (from age 59 to 69 for men; from age 54 to 64 for women)^4^ using the KSA population pyramid to estimate the number of health workers affected by this change.
3. *Providing continuing education for nurses.* To augment the supply of nurses, the KSA has previously implemented bridge programs that provide additional training to those with diploma degrees to equip them to become higher degree nurses [19]. Based on input from local experts from the Saudi Health Council and the World Bank regional office, we assumed that 40 % of nurses with diploma degrees could be trained in such bridge programs (see Appendix C for additional details).
4. *Reallocating health worker budgets.* Drawing from need-based projections for 2030 [7], we used estimated salary amounts from the KSA’s public sector salary scale to illustrate the principle of resource reallocation that demand-side employers can use to alter workforce composition (see Table D1 in Appendix D for details). Given that the MOH is the dominant employer of health workers in the KSA, resources going toward salaries for Saudi physicians in excess of service delivery needs [7] can be redirected toward increasing the demand (or affordability) of Saudi nurses, of whom there are an insufficient number to deliver necessary health services.

## Results

### Health labor market supply

**Fig. 1:**
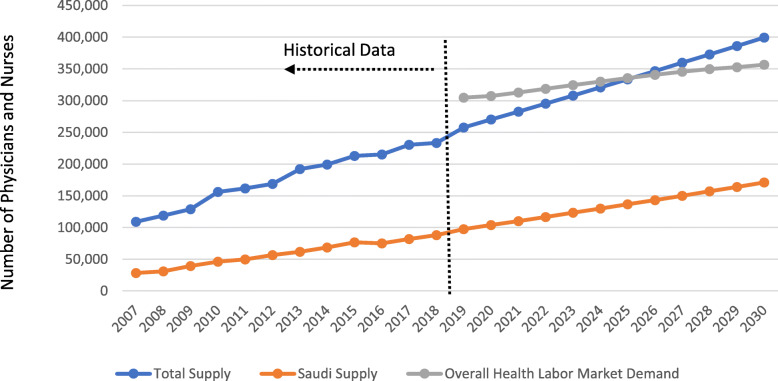
Historical Supply of Health Workers and Their Projected Supply and Demand. Data source: The Kingdom of Saudi Arabia Ministry of Health historical data and original calculations for this publication. Note: ***Overall Demand*** trend indicates the overall health labor market demand in the KSA (including all employers in both the public sector and the private sector); ***Saudi Supply*** of physicians and nurses includes all physicians (all professional categories) and bachelor and advanced nurses (excluding diploma nurses) who are Saudi nationals (rather than foreign migrants); ***Total Supply*** of physicians and nurses includes all physicians (specialist and generalist) and all nurses (diploma, bachelor, and advanced) inclusive of all nationality (Saudi and foreign).

Figure 1 shows the projected supply and demand of physicians and nurses in the KSA to 2030. Overall, the total supply of physicians and nurses (including both Saudi and foreign) will grow from 270,198 in 2020 to 399,354 in 2030 (for physicians: from 92,642 to 138,635, respectively; for nurses: from 177,556 to 260,719, respectively) (Table 1).

**Table. 1:** Projected Supply and Demand for Physicians and Nurses

Cadre	Total Supply		Saudi Supply^a^		Overall Labor Market Demand		Public Sector Demand	
	Number	Density	Number	Density	Number	Density	Number	Density
**Physicians**								
** 2020**	92,642	2.66	19,563	0.56	104,145	2.99	61,533	1.77
**2030**	138,635	3.53	34,914	0.89	120,099	3.05	70,368	1.79
**Nurses**								
**2020**	177,556	5.10	20,744	0.60	203,040	5.83	145,004	4.17
** 2030**	260,719	6.63	33,705	0.86	236,415	6.01	166,184	4.23
**Physicians + Nurses**								
** 2020**	270,198	7.76	40,307	1.16	307,185	8.82	206,537	5.95
**2030**	399,354	10.16	68,619	1.75	356,514	9.06	236,552	6.02

Data source: Original calculations for this publication. Note: ^a^The Saudi supply of physicians and nurses is FTE-adjusted; the Saudi nurse supply includes only nurses with bachelor’s and advanced degree.

However, when focusing on Saudi workers only, we estimate that in 2030, there will be 34,914 (0.89 per 1,000) Saudi physicians and 33,705 (0.86 per 1,000) Saudi nurses. The percentage of Saudi physicians relative to their foreign counterparts will increase very little—from 25 to 30 %—over this time period. The increase in the percentage of Saudi bachelor and advanced nurses versus total number of nurses is even smaller—from 12 % in 2020 to 13 % in 2030. Foreign physicians and nurses will continue to dominate the workforce in the KSA, assuming no policy intervention.

### Health labor market demand

We estimate that in 2020, 307,185 health workers will be demanded, growing modestly to 356,514 workers (9.07 per 1,000) in 2030. Over this time period, demand for physicians will grow by only 15 % to reach 120,099 (3.05 per 1,000) in 2030; demand for nurses will grow by 16 % to reach 236,415 (6.01 per 1,000) in 2030. Public sector demand will remain steady over time, comprising about 67 % of overall demand (6.02 per 1,000) in 2030, including 59 % of overall demand for physicians (1.79 per 1,000; 70,368 demanded by the public sector) and 70 % of overall demand for nurses (4.23 per 1,000; 166,184 demanded by the public sector). Projections for nurses, however, include all sub-categories of nurses, which may overestimate the demand for nurses—as defined by international standards for the nursing professional scope of work—that the KSA may be able to afford.

### Gaps between supply and demand

Without policy intervention, there is an estimated overall labor market shortage of 36,987 workers in 2020, which will increase by 16 % to yield a surplus of 42,840 health workers in 2030. The shortage of physicians (11,503 in 2020) will transition into a surplus of 18,536 physicians in 2030, but the shortage of nurses will decrease by 5 % (from 25,484 to 24,30). Despite overall market shortages, estimated future supply will exceed the number estimated to be demanded by the public sector for both physicians and nurses.

When counting only the supply of Saudi workers compared with total demand, estimated shortages in 2030 become more severe—287,895 Saudi workers (85,185 physicians and 202,710 nurses)—relative to the overall market situation for all workers. With public sector demand alone, there will be a shortage of 167,933 Saudi workers in 2030. However, in terms of densities, the gap between overall demand and the supply of Saudi workers is projected to narrow by 0.35 per 1,000 population (from 7.67 per 1,000 population in 2020 to or 7.32 per 1,000 population in 2030), suggesting a general improvement over time. Similarly, when considering only public sector demand, shortages of Saudi workers will narrow from 4.77 per 1,000 population in 2020 to 4.27 per 1,000 population in 2030. Notably, while the shortage of Saudi physicians in the public sector may reduce from 41,970 to 35,454 over time, shortages of nurses will increase from 124,260 to 132,479.

### Simulating policy scenarios

**Fig. 2:**
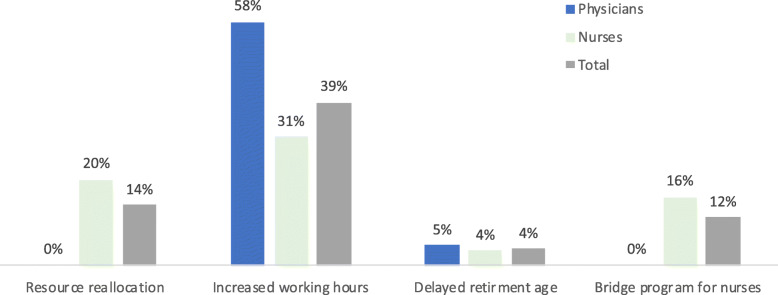
Simulated Effects of Selected Workforce Policies on Reducing Market Shortages. Data source: Original calculations for this publication.

 Figure 2 summarizes the simulated policy scenarios compared with baseline estimates.

1. ***Increasing working hours***: If work hours were increased from five to eight hours per day for Saudi workers, the 2030 FTE supply of Saudi workers would increase by 112,184 (49,631 physicians, 62,553 bachelor and advanced nurses), reducing the shortage of Saudi workers by 39 % (58 % for physicians, 31 % for nurses) in the overall market, and by 67 % in the public sector in which the shortage of physicians could be eliminated and that for nurses reduced by 47 %.
2. ***Improving retention.*** Delaying retirement for 10 years for Saudi workers will add 11,713 FTE Saudi workers (4,215 physicians, 7,498 bachelor and advanced nurses) to the workforce in 2030 (see Tables C3 and C4 in Appendix C), which reduces the shortage of Saudi workers by 4 % (5 % for physicians, 4 % for nurses) in the overall market, and by 7 % (12 % for physicians, 6 % for nurses) in the public sector.
3. ***Continuing education for diploma nurses.*** If 40 % of diploma nurses receive continued training, this will add 33,267 to the Saudi nursing cadre in 2030 (see Table C5 in Appendix C), reducing nursing shortages by 16 % overall and 25 % in the public sector.
4. ***Reallocating health worker budgets.*** At baseline, reallocating public sector salary support from Saudi physicians in excess of what is needed to deliver health services (per epidemiologic need-based modeling [7]) toward Saudi bachelor and advanced nurses can employ 41,539 nurses in 2030, reducing nursing shortages by 20 % overall and 31 % in the public sector.

## Discussion

With a health system in transition, the government of the KSA plans to build a more resilient workforce with less reliance on foreign health workers. Our projected demand and supply estimates of physicians and nurses in the KSA indicate that the overall demand and supply of health workers is relatively balanced. By 2030, there will be a modest surplus of about 42,840 physicians and bachelor and advanced nurses in 2030. Although we estimate the total supply of physicians and nurses to be 10.16 per 1,000 in 2030, because only about 17 % of these workers are Saudi nationals, there will be only 0.89 Saudi physicians and 2.86 Saudi nurses per 1,000, yielding a substantial shortage of workers who are Saudi nationals to meet anticipated demand.

### Policy Scenario Implications

While market shortages for Saudi health workers will persist, among the policy scenarios considered, increasing working hours from five to eight hours per day had the largest impact on reducing shortages. Delaying retirement age is expected to have the smallest impact. Bridge programs that provide additional training to 40 % of diploma nurses will more than double the projected nurse workforce supply but will not eliminate nursing shortages.

Overall demand-based shortages of Saudi workers may still remain even after considering different supply-side policies; however, reallocating resources for recruiting workers among demand-side employers may substantially improve the adequacy of the health workforce toward more optimally delivering health services. In particular, specific epidemiological modeling of health workforce needs for the KSA show that the number of Saudi physicians exceeds what is needed for service delivery, but the number of Saudi nurses is insufficient [7]. Reallocating public sector salary support from the number of Saudi physicians that exceed what is required to deliver health services toward Saudi nurses can substantially reduce market shortages of these nurses. At the same time, such reallocation will also completely eliminate the need-based gap for nurses to deliver health services in 2030. Hence, for the Saudi nurse cadre specifically, a combination of demand- and supply-side policies may be needed to address both market-based shortages and service delivery needs. For example, relaunching the bridge programs may be a useful tool while at the same time redeploying financial resources toward hiring those additional nurses into the health system.

### Limitations

Results from our analyses include several caveats. Projecting the number of health workers involves predictive uncertainty; as such, the resulting estimates should be interpreted as only indicative. To that end, we have reported aggregated estimates, which are more likely to be comparable and to absorb the margins of error associated with individual estimates for subnational data points. We are also limited by the available data for health workers and government spending. Physician data contain a small proportion of dentists, which could not be disaggregated, but this similarly affects both demand and supply estimates. Data on private sector spending were not available; thus, this model implicitly assumes that future labor market demand for health workers is driven by government spending. Collecting additional private sector spending, including out-of-pocket expenditures, can inform a more tailored demand model that may better reflect anticipated resource diversification and mobilization reforms in Saudi Arabia. In supply-side policy scenarios, we assumed that working hours directly relate to workers’ relative productivity, which neglects efficiency factors that may be relevant to the number of patients each provider can care for during each working day. Delayed retirement assumed that retained health workers continued in their position with the same productivity rather than advancing in their careers. None of these scenarios account for time to implement the policy. Whether or not workers have the willingness or the incentives to respond to policy changes may influence the outcomes of policy scenarios. Health workers’ adherence to policy changes were not modeled in this study due to data limitations. This important topic should be evaluated and taken into considerations in future studies.

## Conclusions

We adapted methods previously used in other studies for cross-country global workforce projections for the KSA labor market for health workers to inform strategic workforce planning. Despite the projected overall balance in the labor market for health workers and sufficient densities to meet health service delivery goals [7] in 2030, we find persistent and severe gaps in the Saudi national workforce that will limit progress toward health system resiliency and self-sufficiency in the KSA. To ameliorate the situation, both supply- and demand-side policy interventions should be considered, prioritizing those that increase productivity among Saudi health workers, enhance training for nurses, and strategically redeploy financial resources toward employing these workers. Concomitant interventions will also likely be needed to ensure that poorer segments of the populations are protected. Public resources should be prioritized for these interventions while private sector resources are increasingly mobilized in line with overall macroeconomic restructuring.

## Supplementary Information

**Additional file 1:**

## Data Availability

The datasets generated and/or analyzed during the current study are available in the Kingdom of Saudi Arabia, Ministry of Health data repository, https://www.moh.gov.sa/en/Ministry/Statistics/book/Pages/default.aspx.

## Footnotes

2 Other public sector employers of health workers include the National Guard, the Ministry of Defense, the Ministry of Interior, and King Faisal Specialist Hospital, among others.

3 Saudi workers, especially those employed in the public sector, typically have the working hours of 9am to 3pm, including a one-hour lunch. When comparing this to an FTE eight-hour workday, a 63 % (five hours/eight hours) maximum productivity was estimated for public sector workers.

4 In August 2019, retirement age was changed to age 60 for both men and women. However, because the data used for this study are from the period before the implementation of this policy, we based our simulation on the pre-2019 retirement ages.
