# Correction to: Projecting health labor market dynamics for a health system in transition: planning for a resilient health workforce in Saudi Arabia

**DOI:** 10.1186/s12992-021-00773-6

**Published:** 2021-11-01

**Authors:** Tracy Kuo Lin, Tim A. Bruckner, Taghred Alghaith, Mariam M. Hamza, Mohammed Alluhidan, Christopher H. Herbst, Hussah Alghodaier, Adwa Alamri, Rana Saber, Nahar Alazemi, Jenny X. Liu

**Affiliations:** 1 Institute for Health & Aging, Department of Social and Behavioral Sciences, University of California, 490 Illinois St, San Francisco, CA 94158 USA; 2 Center for Population, Inequality, and Policy, University of California, Irvine, USA; 3 Saudi Health Council, Riyadh, Saudi Arabia; 4 World Bank, Washington, DC USA; 5 Division of Health Research, Lancaster University, Lancaster, UK

**Correction to: Global Health 17, 105 (2021)**

**https://doi.org/10.1186/s12992-021-00747-8**

Following publication of the original article [1], the authors flagged errors in the Introduction and the Discussion of their article.

Namely, in the Projecting Demand section of the Background, the ‘ξpt predictor variables were lagged’ had been written in place of ‘Predictor variables were lagged’. While in the opening paragraph of the Discussion ‘2.86 Saudi nurses per 1,000’ had been erroneously written instead of ‘0.86 Saudi nurses per 1,000’.

The original article has since been updated to amend these errors.

## References

[1] Lin TK, Bruckner TA, Alghaith T, Hamza MM, Alluhidan M, Herbst CH, Alghodaier H, Alamri A, Saber R, Alazemi N, Liu JX (2021). Projecting health labor market dynamics for a health system in transition: planning for a resilient health workforce in Saudi Arabia. Glob Health.

