# Supplementary material for: Projecting health labor market dynamics for a health system in transition: planning for a resilient health workforce in Saudi Arabia
Source: Global Health. 2021 Sep 14;17:105. doi: 10.1186/s12992-021-00747-8 (PMC8439018; doi:10.1186/s12992-021-00747-8)
Supplement: Supplementary file 1 — Additional file 1: [file 12992_2021_747_MOESM1_ESM.docx]

**Appendices for Projecting Health Workforce Labor Market Demand for a Health System in Transition: Health Workforce Planning for the Kingdom of Saudi Arabia**

**Appendix A**

| **Table A1 Data Source for economic modeling** | | | |
| --- | --- | --- | --- |
| **Indicator** | **Unit** | **Years** | **Source** |
| Number of physicians and nurses | Region | 2007-2018 | Kingdom of Saudi Arabia Ministry of Health (MoH)^[[1]](#footnote-1)^ |
| Number of physicians and nurses, by nationality (Saudi versus foreign) | Region | 2007-2018 |  |
| Number of physicians and nurses, by gender | Region | 2007-2018 |  |
| Number of physicians and nurses, by facility type (hospital or primary care) | Region | 2007-2018 |  |
| Number of physicians and nurses, by sector (MoH/ or private) | Region | 2007-2018 |  |
| Number of physicians and nurses in the public sector, by MoH and non-MoH employers | National | 2007-2018 |  |
| Population | Region | 2007-2018 |  |
| Total population and population projection | National | 2007-2030 | United Nations, Department of Economic and Social Affairs Population Division^[[2]](#footnote-2)^ |
| Future estimated population | National | 2019-2030 | United Nations, Department of Economic and Social Affairs, Population Division |
| MoH Health expenditure | Provincial | 2009-2018 | BOOST Data |
| Total government expenditure | Provincial | 2009-2018 | BOOST Data |
| Estimated future GDP PPP | National | 2019-2030 | World Bank |

**Appendix B**

| **Table B1: Labor Market Demand Projection Summary** | | | |
| --- | --- | --- | --- |
| **Sector** | **Historical data** | **Methodology** | **Projected Values** |
| Public Sector – MoH | MoH physicians and nurses | Using MoH health expenditure, Total government expenditure as predictors for physician and nurse densities | 1. Labor market demand for physicians in the KSA (entire public and private sector), from 2020 to 2030 2. Labor market demand for physicians in the public sector in KSA (MOH and non-MOH employers), from 2020 to 2030 3. Labor market demand for nurses in the KSA (entire public and private sector), from 2020 to 2030 4. Labor market demand for nurses in the public sector in KSA (MOH and non-MOH employers), from 2020 to 2030 |
| Public Sector – non-MOH | Percentage of non-MoH works out of total public sector | Using historical data to predict the percentage of non-MoH works out of total public sector in the future |  |
| Private Sector | Private sector physicians and nurses | Using MoH health expenditure, Total government expenditure as predictors for physician and nurse densities |  |

The projection methodology adopted a stepwise approach described below.

*Step 1. Determination of economic drivers*

Although health expenditure is considered to be a more ideal indicator for demand, because of a lack of data, previous studies on demand for health workers relied on proxy indicators. Studies have shown that indicators of GDP or national income are the best predictors of health expenditures, of which labor is the principal component (Cooper, Getzen, and Laud 2003; Getzen 1990; Newhouse 1977). The demand model projection for global health worker labor market demand by Liu et al. (2017) similarly used per capita indicators of GDP (Liu et al. 2016), but also included household out-of-pocket (OOP) health expenditures as a proxy measure of the generosity of health insurance coverage, as well as the size of the population aged 65 or over as an indicator of the demographic effect of population aging and ensuing demand for health care services utilized at older ages (Cooper et al. 2002).

Using historical data on physician and nurse densities, health expenditure per capita, and total governmental expenditure per capita, the relationship between the economic drivers and health worker densities were estimated using a generalized linear model (GLM). All variables were transformed into logs. To avoid endogeneity, health expenditure per capita and total governmental expenditure were lagged up to three years to allow time for such factors to work through the economy and affect the labor market, as other authors have done in previous projection exercises (Getzen 19990; Scheffler et al. 2008). A stepwise approach was used to select the specific combination of year lags that maximized the predictive power of each variable. Lagged variables that achieved a minimum 1 percent level of significance after repeated iteration were kept within the model, resulting in the following optimal model for physicians and nurses, respectively:

ln(MOH & private sector *physicians per 1,000 population_it_*) = α_0_ + α_1_*ln(*health expenditure per capita_it-1_*) + *μ_p_* + *ξ_it_* (Eq 1)

ln(MOH & private sector *nurses per 1,000 population_it_*) = β_0_ + β_1_*ln(*health expenditure per capita_it-1_*) + β_2_*ln(*total governmental expenditure per capita_it-4_*) + *μ_p_* + *ξ_it_* (Eq 2)

where *μ_p_* represents a vector of provincial fixed effects, *ξ_it_* is the disturbance terms, and β coefficients are unknown parameters to be estimated from the model. Provincial fixed effects *μ_p_* were included to account for time-invariant unobservable heterogeneity (that is, not captured by available data) across provinces (for example, average distance to closest hospitals).

While specification testing showed that the optimal model for physicians included only one economic predictor whereas the model for nurses included two predictors, the final predictive model employed both health expenditures and total government expenditures, each lagged one year for several reasons. First, this ensured that the models used to predict both physicians and nurses rest on the same theoretical assumptions regarding the economic drivers. Second, comparison of Akaike Information Criterion (AIC) and Bayesian Information Criterion (BIC) showed that including both economic predictor variables produced nearly the same test statistic value (see table 5A.3) as the model using only one predictor variable (as in Equation 1). Third, because the nursing cadre is larger than that for physicians in KSA, the model projections for nurses will have a larger substantive impact on resource mobilization. Model 1 in table 5A.3 included the coefficient using one predictor variable (Equation 1). Given that both the predictor and outcome variables have been log-transformed, the results indicated that 1 percent increase in health expenditure per capita is associated with a 20 percent increase in physicians per 1,000 population. Model 2 in Table 5.1.3 included the coefficients for physicians per 1,000 using both predictor variable. The results indicated that 1 percent increase in health expenditure per capita is associated with approximately 16 percent increase in physicians per 1,000 population; 1 percent total government expenditure is associated with 3 percent increase in physicians per 1,000, but this predictor is not statistically significant. Model 3 in table 5A.3 presented the results for nurses per 1,000 using two predictors (Equation 2). The results indicated that a 1 percent increase in health expenditure per capita is associated with approximately 13 percent increase in nurses per 1,000 and 1 percent increase in total governmental expenditure is associated with approximately 7 percent increase in nurses per 1,000 population.

| **Table B2 Model fit (using historical data)** | | | |
| --- | --- | --- | --- |
|  | **(1)** | **(2)** | **(3)** |
| **Variable** | **Ln(MOH & private sector physicians per 1,000 population_it_)** | **Ln(MOH & private sector physicians per 1,000 population_it_)** | **Ln(MOH & private sector nurses per 1,000 population_it_)** |
|  |  |  |  |
| Ln(Health Expenditure per capita)_t−1_ | 0.199*** | 0.146*** | 0.129** |
|  | (0.0306) | (0.0482) | (0.0551) |
| Ln(Total Governmental Expenditure per capita) |  | 0.0305 | 0.0653*** |
|  |  | (0.0213) | (0.0244) |
| Constant | −0.318 | −0.141 | 0.409 |
|  | (0.207) | (0.241) | (0.275) |
| AIC | −1.473512 | −1.476291 | −1.206426 |
| BIC | −489.2684 | −484.5306 | −484.1554 |
|  |  |  |  |
| Observations | 117 | 117 | 117 |

*Source:* Original calculations for this publication.

*Note:* AIC = Akaike Information Criterion; BIC = Bayesian Information Criterion.

** *p* < .05 *** *p* < .001

*Step 2. Projecting future value of predictors*

Future values of health expenditure are needed as input parameters into the demand model projections to predict future values of physicians per 1,000 population and nurses per 1,000 population. To project future values of health expenditure per capita, seven different models were tested:

1. For each province *p*, a moving average for 18 prior periods without weights was estimated:

${health expenditure}_{pt}=\frac{1}{12}\sum_{x=1}^{11} {health expenditure}_{p,t-x}$ (Eq 3)

A total of 12 previous periods was chosen to maximize the number of prior year observations possible to base projections off of (for example, the projection for 2030 needs at least 12 lags to use prior information from 2018).

1. For each province *p*, a moving average for 10 prior periods with weights was estimated, with the more distant observations progressively receiving smaller weights:

${health expenditure}_{pt}=\frac{1}{10}\sum_{x=1}^{10} {\left( 10-x \right)health expenditure}_{p,t-x}$ (Eq 4)

1. For each province *p*, double-exponential smoothing over 10 prior periods was applied:

*S_pt_*^[2]^ = α*S_pt_* + (1−α)*S_p,t−1_*^[2]^, (Eq 5)

where *S_pt_*^[2]^ is the smoothed original series from *S_pt_* = α*X_pt_* + (1−α)*S_p,t−1_*, and α is the smoothing parameter estimated by minimizes the in-sample sum-of-squared predicted errors; *X_pt_* is the original series.

1. For each province *p*, the following regression was applied, where *β* represents the yearly difference in health expenditure over the previous year, and ε*_pt_* is a random error term:

*health expenditure_pt_* = *α* + *βYear trend_pt_* + ε*_pt_* (Eq 6)

1. For each province *p*, the following regression that additionally accounts for non-linearities in the time trend of health spending was estimated:

*health expenditure_pt_* = *α* + *β*_1_*Year trend_pt_* + *β*_2_(*Year trend_pt_)*^2^ + ε*_pt_* (Eq 7)

1. For all province *p*, the following pooled regression that includes province fixed effects (*γ_p_*) as well as a flexible time trend was estimated:

*health expenditure_pt_* = *α_c_* + *β*_1_*Year trend_pt_* + *β*_2_(*Year trend_pt_*)^2^ + *γ_pt_*+ *ε_pt_* (Eq 8)

1. This model uses the same regression for Equation 3 but corrected for autoregressive serial correlation.

Each model specification was first estimated using the initialization dataset, using the lag structure from Equation 1 and Equation 2. The estimated parameters were then applied to the data set to obtain predicted values for 2009 through 2018. These predicted values were then compared with actual data from 2009 to 2018. To assess the fit of each model, the mean square root of the squared error is again calculated in Equation 9; the results are displayed in table 5A.4 for each of the models tested.

Mean error = $\frac{1}{PT}\sum\sqrt{{\hat{{(health spending}_{pt}}-{health spending}_{pt})}^{2}}$ (Eq 9)

| **Table B3 Mean errors for health spending per capita projection models** | | | |
| --- | --- | --- | --- |
| **Model** | ***N = P* × *T*** | **Mean** | **Standard Deviation** |
| 1 | 117 | 46,038.29 | 96,127.89 |
| 2 | 117 | 38,526.09 | 84,861.62 |
| **3** | **130** | **12,095.64** | **30,076.51** |
| 4 | 130 | 73,775.19 | 99,767.52 |
| 5 | 130 | 73,336.20 | 97,681.25 |
| **6** | **130** | **15,855.27** | **36,518.44** |
| 7 | 130 | 23,363.77 | 58,253.15 |

*Source:* Original calculations for this publication.

*Note:* N = number; P = province; T = time period (year).

Models 3 and 6 (table 5A.4) were found to have the smallest prediction error. Moreover, the mean of the predictions from Models 3 and 6 were evaluated to see if prediction accuracy was improved (Makridakis, Wheelwright, and Hyndman 2008). To further improve prediction accuracy, the optimal method (which minimizes the square root of the squared error) for each province was identified for inclusion (instead of relying on one method for all provinces). For example, Model 3 may minimize prediction errors in province A, but weighted average may minimize error in province B. Rather than force one method on all provinces uniformly, the optimal model selection was allowed to be province specific. The results from this approach indicated that Model 3 generated the smallest errors across all provinces; therefore, Model 3 was used for all provinces.

The same steps were used for predicting future values of both health spending per capita and total governmental spending per capita. Table 5A.5 displays the mean errors associated with the models tested for predicting total spending per capita. These results show that Model 3 was the method found to have the smallest prediction error.

| **Table B4 Mean errors for total spending per capita projection models** | | | |
| --- | --- | --- | --- |
| **Model** | ***N = P × T*** | **Mean** | **Standard Deviation** |
| 1 | 117 | 25321.35 | 37006.60 |
| 2 | 117 | 19959.16 | 28690.76 |
| **3** | **130** | **1551.467** | **3558.455** |
| 4 | 130 | 32036.45 | 41569.14 |
| 5 | 130 | 31875.68 | 42551.93 |
| 6 | 130 | 4135.892 | 6013.235 |
| 7 | 130 | 8129.914 | 13696.85 |

*Source:* Original calculations for this publication.

*Step 3. Future labor market demand for physicians and nurses*

The predicted values for health expenditure per capita and total governmental expenditure per capita were then used as inputs into the overall demand model (Equation 2) along with estimated coefficients (table 5A.3) to estimate the density of physicians and nurses in each province-year from 2020 to 2030. Predicted values of logged physician and nurse densities from this model were then transformed with an antilog and multiplied by a correction factor ($e^{\sigma^{2}/2}$) to account for the skewed distribution. The density for each cadre in each province-year were multiplied by the provincial population to calculate the labor market demand of health workers in the province. The provincial demands for physicians and nurses were then summed by year to calculate the national labor market demand for physicians and nurses from 2020 to 2030.

*Step 4. Overall health labor market demand for physicians and nurses in KSA*

Additional steps were taken to project the total number of public sector workers, incorporating the supply of physicians and nurses in the public sector but who were not employed by the MOH. Because the yearly data on the number of total public sector workers at the provincial level were not available, national-level data on the number of public sector health workers in the MOH and outside the MOH were used. The national percentages of physicians and nurses working for the MOH out of the total public sector workers were projected into the future. The projected percentages of MOH physicians and nurses out of total public sector physicians and nurses were then used to calculate the estimated national number of health workers, including all public sector employers, from 2020 to 2030 (table 5A.6).

| Table B5 Projected labor market demand for physicians and nurses | | | | | | |
| --- | --- | --- | --- | --- | --- | --- |
|  | **Physicians** | | **Nurses** | | **Physicians and Nurses** | |
| Year | **Number** | **Density per 1,000 population** | **Number** | **Density per 1,000 population** | **Number** | **Density per 1,000** |
| 2020 | 104,145 | 2.99 | 203,040 | 5.83 | 307,185 | 8.82 |
| 2021 | 106,010 | 3.00 | 206,819 | 5.85 | 312,830 | 8.85 |
| 2022 | 107,936 | 3.01 | 210,779 | 5.88 | 318,716 | 8.89 |
| 2023 | 109,806 | 3.02 | 214,632 | 5.91 | 324,438 | 8.93 |
| 2024 | 111,649 | 3.03 | 218,447 | 5.94 | 330,096 | 8.97 |
| 2025 | 113,435 | 3.05 | 222,168 | 5.96 | 335,604 | 9.01 |
| 2026 | 115,054 | 3.05 | 225,586 | 5.99 | 340,640 | 9.04 |
| 2027 | 116,602 | 3.06 | 228,858 | 6.00 | 345,460 | 9.06 |
| 2028 | 118,040 | 3.06 | 231,856 | 6.02 | 349,896 | 9.08 |
| 2029 | 118,975 | 3.06 | 233,868 | 6.01 | 352,843 | 9.06 |
| 2030 | 120,099 | 3.05 | 236,415 | 6.01 | 356,514 | 9.07 |

*Source:* Original calculations for this publication.

*Step 5. Projected numbers of physicians and nurses (baseline)*

*Step 5. Public sector health labor market demand for physicians and nurses in KSA*

To allow for comparison with the projection for the overall health labor market demand in KSA, additional estimations were conducted to project public sector–specific demand. While past literature (Liu et al. 2016) employed demand predictors that captured economy-wide spending, the analysis here is limited to data on MOH and government spending only; no private sector demand-side predictors were available in KSA. Concomitantly, the government-specific spending data are accompanied by historical data for MOH workers only (excluding health workers in the public sector who were employed by non-MOH employers). As such, the predictions for the overall health labor market demand in KSA were being driven entirely by trends in the public sector spending. The same predictors were used to predict the demand for MOH workers when estimating public sector–specific demand for workers. As expected, the overall health labor market demand and public sector–specific labor market demand yielded parallel lines, with public sector–specific demand projecting a lower number. This is a statistical product of the data limitations. Without any indicators of private sector growth, this assumption is necessary. Nevertheless, this projection may reflect the health labor market demand in a baseline scenario with no policy intervention.

Following the methodology outlined for projecting the overall health labor market demand in KSA (in step 1), a stepwise approach was used to select the specific combination of year lags that maximized the predictive power of each variable. Lagged variables that achieved a minimum 1 percent level of significance after repeated iteration were kept within the model, resulting in the following optimal model for physicians and nurses, respectively:

ln(MOH *physicians per 1000 population_it_*) = α_0_ + α_1_*ln(*health expenditure per capita_it-1_*) + *μ_p_* + *ξ_it_* (Eq 10)

and

ln(MOH *nurses per 1000 population_it_*) = β_0_ + β_1_*ln(*health expenditure per capita_it-1_*) + β_2_*ln(*total governmental expenditure per capita_it-4_*) + *μ_p_* + *ξ_it_* (Eq 11)

where *μ_p_* represents a vector of provincial fixed effects, *ξ_it_* is the disturbance terms, and β coefficients are unknown parameters to be estimated from the model. Provincial fixed effects *μ_p_* were included to account for time-invariant unobservable heterogeneity (that is, not captured by available data) across provinces (for example, average distance to closest hospitals).

Similar to the specification testing results for the model including MOH and private health workers, the specification testing here showed that the optimal model for physicians included only one economic predictor, whereas the model for nurses included two predictors. Following the rationale for previous models, the final model predictive model for MOH workers employed both health expenditures and total government expenditures, each lagged one year for several reasons. First, this ensured that the models used to predict both physicians and nurses rest on the same theoretical assumptions regarding the economic drivers. Second, comparison of Akaike Information Criterion (AIC) and Bayesian Information Criterion (BIC) showed that including both economic predictor variables produced nearly the same [test stat value] as the model (see Table 5.1.7) using only one predictor variable (as in Equation 10). Third, because the nursing cadre is larger than that for physicians in the public sector in KSA, the model projections for nurses will have a larger substantive impact on resource mobilization. Model 1 in table 5A.7 included the coefficient using one predictor variable (Equation 10). Model 2 in table 5A.7 included the coefficients for physicians per 1,000 using both predictor variables. Model 3 in table 5A.1.7 presented the results for nurses per 1,000 using two predictors (Equation 11).

| **Table B6: Model Fit (using historical data)** | | | |
| --- | --- | --- | --- |
|  | **(1)** | **(2)** | **(3)** |
| **Variable** | **Ln(MOH Physicians per 1,000 population_it_)** | **Ln(MOH Physicians per 1,000 population_it_)** | **Ln(MOH Nurses per 1,000 population_it_)** |
|  |  |  |  |
| **Ln(Health Expenditure per capita)_t-1_** | 0.179*** | 0.123*** | 0.107** |
|  | (0.027) | (0.0428) | (0.0538) |
| **Ln(Total Governmental Expenditure per capita)** |  | 0.0316* | 0.0654*** |
|  |  | (0.018) | (0.0238) |
| **Constant** | −0.313 | −0.129 | 0.498 |
|  | (0.184) | (0.214) | (0.269) |
| **AIC** | −1.702575 | −1.712543 | −1.252813 |
| **BIC** | −489.5215 | −484.7854 | −484.2273 |
|  |  |  |  |
| **Observations** | 117 | 117 | 117 |

*Source:* Original calculations for this publication.

*Note:* AIC = Akaike Information Criterion; BIC = Bayesian Information Criterion.

The future values of the predictors health spending per capita and total governmental expenditure per capital, which were projected following the steps outlined in step 2 of this annex, were then used as inputs into the public sector demand model (Equation 11) along with estimated coefficients (table 5A.7) to estimate the density of physicians and nurses in each province-year from 2019 to 2030. Predicted values of logged MOH physician and MOH nurse densities from this model were then transformed with an antilog and multiplied by a correction factor ($e^{\sigma^{2}/2}$) to account for the skewed distribution. The density for each cadre in each province-year were multiplied by the provincial population to calculate the labor market demand of health workers in province. The provincial demand for physicians and nurses were then summed by year to calculate the national labor market demand for physicians and nurses from 2019 to 2030.

To estimate public sector–specific demand for health workers, the projected national number of MOH physicians and MOH nurses were used as the numerator and the percentages of MOH workers out of total public sector workers (generated step 4 of this annex) were used as the denominator. Table 5A.8 includes the estimated public sector health labor market demand for physicians and nurses.

| Table B7: Projected public sector labor market demand for physicians and nurses | | | | | | |
| --- | --- | --- | --- | --- | --- | --- |
|  | **Physicians** | | **Nurses** | | **Physicians and Nurses** | |
| Year | **Number** | **Density per 1,000 population** | **Number** | **Density per 1,000** | **Number** | **Density per 1,000 population** |
| 2020 | 61,533 | 2.99 | 145,004 | 4.17 | 206,537 | 5.93 |
| 2021 | 62,182 | 3.00 | 145,271 | 4.11 | 207,454 | 5.87 |
| 2022 | 63,287 | 3.01 | 148,059 | 4.13 | 211,346 | 5.90 |
| 2023 | 64,358 | 3.02 | 150,772 | 4.15 | 215,130 | 5.92 |
| 2024 | 65,413 | 3.03 | 153,456 | 4.17 | 218,869 | 5.95 |
| 2025 | 66,435 | 3.05 | 156,070 | 4.19 | 222,505 | 5.97 |
| 2026 | 67,373 | 3.05 | 158,487 | 4.21 | 225,860 | 5.99 |
| 2027 | 68,275 | 3.06 | 160,808 | 4.22 | 229,083 | 6.01 |
| 2028 | 69,118 | 3.06 | 162,937 | 4.23 | 232,055 | 6.02 |
| 2029 | 69,698 | 3.06 | 164,357 | 4.22 | 234,055 | 6.01 |
| 2030 | 70,368 | 3.05 | 166,184 | 4.23 | 236,552 | 6.02 |

*Source:* Original calculations for this publication.

*Step 6. Projected numbers of physicians and nurses (GDP growth rate scenario)*

A sensitivity analysis was conducted assuming health expenditure increases with estimated GDP growth rate. The projected numbers are presented in table 5A.7.

| **Table B8 Projected labor market demand for physicians and nurses, assuming health expenditure increases with GDP growth rate** | | | | | | |
| --- | --- | --- | --- | --- | --- | --- |
|  | **Physicians** | | **Nurses** | | **Physicians and Nurses** | |
| **Year** | **Number** | **Density per 1,000 population** | **Number** | **Density per 1,000** | **Number** | **Density per 1,000 population** |
| **2020** | 104,092 | 2.99 | 202,887 | 5.83 | 306,980 | 8.82 |
| **2021** | 105,968 | 3.00 | 206,669 | 5.85 | 312,637 | 8.85 |
| **2022** | 107,968 | 3.01 | 210,728 | 5.88 | 318,381 | 8.89 |
| **2023** | 109,831 | 3.02 | 214,550 | 5.91 | 324,000 | 8.93 |
| **2024** | 111,668 | 3.03 | 218,332 | 5.93 | 330,000 | 8.97 |
| **2025** | 113,449 | 3.05 | 222,026 | 5.96 | 335,475 | 9.01 |
| **2026** | 115,052 | 3.05 | 225,414 | 5.98 | 340,466 | 9.03 |
| **2027** | 116,600 | 3.06 | 228,683 | 6.00 | 345,283 | 9.06 |
| **2028** | 118,038 | 3.06 | 231,678 | 6.01 | 349,716 | 9.08 |
| **2029** | 118,992 | 3.06 | 233,777 | 6.00 | 352,768 | 9.06 |
| **2030** | 120,131 | 3.06 | 236,338 | 6.01 | 356,519 | 9.07 |

**Appendix C**

***Simulated scenarios for relative productivity***

To capture the status quo health workforce supply and allow for comparison with estimated changes in the supply of health workers associated with policy interventions, the projection additionally incorporated relative worker productivity. The projection adjusted for relative productivity of Saudi workers using a five-hour workday (9am to 3pm, including a one-hour lunch) as compared with a full eight-hour workday. The status quo productivity was estimated to be 63 percent (five hours/eight hours) of maximum productivity. The projected supply was multiplied by 63 percent to generate the status quo health workforce supply. Simulated scenarios evaluated the impact on workforce supply with 80 percent relative productivity (six-and-a-half hours/eight hours) and 100 percent (full eight hours). The projection included all Saudi workers in the public sector and the private sector. The FTE supply of physicians under different scenarios is presented in table 1 and the FTE supply of nurses under different scenarios is presented in table 2.

**Table C1 FTE supply of Saudi physicians (including generalists and specialists)**

| **Year** | **Baseline** | **Simulated Scenarios** | |
| --- | --- | --- | --- |
|  | **63% Relative Productivity** | **80% Relative Productivity** | **100% Relative Productivity** |
| **2020** | 19,563 | 24,842 | 31,053 |
| **2021** | 21,011 | 26,681 | 33,351 |
| **2022** | 22,473 | 28,537 | 35,671 |
| **2023** | 23,953 | 30,417 | 38,021 |
| **2024** | 25,452 | 32,320 | 40,401 |
| **2025** | 26,971 | 34,249 | 42,811 |
| **2026** | 28,513 | 36,207 | 45,258 |
| **2027** | 30,074 | 38,189 | 47,736 |
| **2028** | 31,657 | 40,200 | 50,250 |
| **2029** | 33,290 | 42,273 | 52,841 |
| **2030** | 34,914 | 44,336 | 55,420 |

*Source:* Original calculations for this publication.

**Table C2 FTE Supply of Saudi nurses (including bachelor and advanced nurses)**

| **Year** | **Baseline** | **Simulated Scenarios** | |
| --- | --- | --- | --- |
|  | **63% Relative Productivity** | **80% Relative Productivity** | **100% Relative Productivity** |
| **2020** | 20,744 | 26,342 | 32,927 |
| **2021** | 21,979 | 27,910 | 34,887 |
| **2022** | 23,224 | 29,491 | 36,864 |
| **2023** | 24,480 | 31,086 | 38,858 |
| **2024** | 25,747 | 32,695 | 40,868 |
| **2025** | 27,028 | 34,321 | 42,902 |
| **2026** | 28,326 | 35,969 | 44,961 |
| **2027** | 29,636 | 37,634 | 47,042 |
| **2028** | 30,965 | 39,320 | 49,150 |
| **2029** | 32,342 | 41,069 | 51,337 |
| **2030** | 33,705 | 42,799 | 53,499 |

*Source:* Original calculations for this publication.

***Retention through delayed retirement***

Scenarios for delayed retirement for health workers were considered. The supply of Saudi workers under scenarios of 5-year delay in retirement and 10-year delay in retirement were simulated and presented in tables 3 and 4.

**Table C3 FTE supply of Saudi physicians (including generalists and specialists)**

| **Year** | **Baseline** | **Simulated Scenarios** | |
| --- | --- | --- | --- |
|  | **No Delay in Retirement** | **5-Year Delay in Retirement** | **10-Year Delay in Retirement** |
| **2020** | 19,563 | 21,321 | 21,919 |
| **2021** | 21,011 | 22,898 | 23,541 |
| **2022** | 22,473 | 24,492 | 25,179 |
| **2023** | 23,953 | 26,105 | 26,838 |
| **2024** | 25,452 | 27,739 | 28,518 |
| **2025** | 26,971 | 29,395 | 30,221 |
| **2026** | 28,513 | 31,076 | 31,950 |
| **2027** | 30,074 | 32,778 | 33,700 |
| **2028** | 31,657 | 34,504 | 35,476 |
| **2029** | 33,290 | 36,284 | 37,307 |
| **2030** | 34,914 | 38,055 | 39,129 |

*Source:* Original calculations for this publication.

**Table C4 FTE supply of Saudi nurses (including bachelor and advanced nurses)**

| **Year** | **Baseline** | **Simulated Scenarios** | |
| --- | --- | --- | --- |
|  | **No Delay in Retirement** | **5-Year Delay in Retirement** | **10-Year Delay in Retirement** |
| **2020** | 20,744 | 24,591 | 25,542 |
| **2021** | 21,979 | 26,009 | 27,025 |
| **2022** | 23,224 | 27,444 | 28,528 |
| **2023** | 24,480 | 28,893 | 30,046 |
| **2024** | 25,747 | 30,358 | 31,583 |
| **2025** | 27,028 | 31,841 | 33,139 |
| **2026** | 28,326 | 33,339 | 34,712 |
| **2027** | 29,636 | 34,855 | 36,306 |
| **2028** | 30,965 | 36,402 | 37,931 |
| **2029** | 32,342 | 37,950 | 39,561 |
| **2030** | 33,705 | 39,509 | 41,203 |

*Source:* Original calculations for this publication.

**Table C5 FTE supply of Saudi nurses (including bachelor and advanced nurses and bridged diploma nurses)**

| **Year** | **Baseline** | **Simulated Scenarios** | |
| --- | --- | --- | --- |
|  | **No Bridge Program**  **(Only Bachelor and Advanced Nurses)** | **20% Diploma Nurses Receive Continue Education (All Nurses)** | **40% Diploma Nurses Receive Continue Education**  **(All Nurses)** |
| **2020** | 20,744 | 32,677 | 44,610 |
| **2021** | 21,979 | 34,592 | 47,205 |
| **2022** | 23,224 | 36,527 | 49,831 |
| **2023** | 24,480 | 38,480 | 52,481 |
| **2024** | 25,747 | 40,452 | 55,158 |
| **2025** | 27,028 | 42,445 | 57,861 |
| **2026** | 28,326 | 44,460 | 60,595 |
| **2027** | 29,636 | 46,497 | 63,357 |
| **2028** | 30,965 | 48,557 | 66,149 |
| **2029** | 32,342 | 50,663 | 68,985 |
| **2030** | 33,705 | 52,765 | 71,826 |

*Source:* Original calculations for this publication.

**Appendix D**

The average salary of public sector physicians and average salary of public sector nurses presented in table 1 were estimated using the following assumptions:

1. The ranges of physician and nurse wages are assumed to be the same for both Saudi and foreign physicians in the public sector.
2. The health worker wage bill is from 2018 and is assumed to remain unchanged into the future.

**Table D1 Average physician and nurse wage bills and overall public sector wage costs**

| **Cadre** | **Minimum Salary^a^** | **Maximum Salary^a^** | **Average Salary^a^** | **Number of Workers in the Public Sector 2018** | **Total Estimated Cost in 2018^a^** | **Estimated Resources for Wage Bill In 2030^e^** |
| --- | --- | --- | --- | --- | --- | --- |
| **Physicians** | 14,950^b^ | 39,040^b^ | 26,995 | 65,455 | 1,766,957,725 | 2,828,956,247 |
| **Nurses** | 10,400^c^ | 27,455^d^ | 18,928 | 46,378 | 875,949,984 | 1,402,424,146 |
| **Total** |  |  |  |  |  | 4,231,380,393 |
|  | | | | | | |

*Source:* Original calculations for this publication.

*Note:* a Per month in SARs.

b Data are taken from MOH 2013.

c Minimum: SAR 8,000/month + 30% benefits = SAR 10,400/month

d Maximum: SAR 21,119/month + 30% benefits = SAR 27,455/month

e This assumes the wage bill increases with the average GDP growth rate of 1.04 percent.

In scenarios where there is one surplus cadre, the surplus number of workers were multiplied by the average salary to determine the additional resource that can be reallocated to the other cadre. The estimated numbers are presented in table 2.

**Table D2 Summary of the net effects of simulated scenarios compared with baseline model estimates, 2030**

| **Area of Impact** | **Scenario** | **Change in FTE-Equivalent Workers** | **Need-Based Shortage/Surplus*** | | **Demand-Based Shortage/Surplus*** | |
| --- | --- | --- | --- | --- | --- | --- |
|  |  |  | **Physicians** | **Nurses** | **Physicians** | **Nurses** |
| **Supply** | Increase work hours from 5 to 8 hours | +20,506 physicians  +19,794 nurses | +49,631 | −15,900 | −64,680 | −182,916 |
|  | Delaying retirement age by 10 years | +4,215 physicians  +7,498 nurses | +33,340 | −28,196 | −80,971 | −195,213 |

*Source:* Original calculations for this publication.

*Note:* Numbers followed by * are baseline gaps. Demand-based gaps are not impacted by need-based policy; the number presented here is the baseline demand gap.  **“+”** indicates surplus; “−**“** indicates shortage.

1. Ministry of Health, Kingdom of Saudi Arabia. Statistical Yearbook. 2018. [Accessed 2019 Dec 20] Available from: https://www.moh.gov.sa/en/Ministry/Statistics/book/Pages/default.aspx [source information from Mariam] [↑](#footnote-ref-1)
2. United Nations, Department of Economic and Social Affairs, Population Division. World Population Prospects 2019. [Accessed 2019 Nov 10] Available from: https://www.un.org/en/development/desa/population/publications/database/index.asp [↑](#footnote-ref-2)
